# Comparative Genomic Analysis of Primary and Synchronous Metastatic Colorectal Cancers

**DOI:** 10.1371/journal.pone.0090459

**Published:** 2014-03-05

**Authors:** Sun Young Lee, Farhan Haq, Deokhoon Kim, Cui Jun, Hui-Jong Jo, Sung-Min Ahn, Won-Suk Lee

**Affiliations:** 1 Lee Gil Ya Cancer and Diabetes Institute, Gachon University, Incheon, Korea; 2 Center for Cancer Genome Discovery, Asan Institute for Life Science, University of Ulsan College of Medicine, Asan Medical Center, Seoul, South Korea; 3 Department of Oncology, University of Ulsan College of Medicine, Asan Medical Center, Seoul, Korea; 4 Department of Biomedical Informatics, University of Ulsan College of Medicine, Asan Medical Center, Seoul, South Korea; 5 Department of Surgery, Gil Medical Center, Gachon University, Incheon, Korea; University Medical Center Hamburg-Eppendorf, Germany

## Abstract

Approximately 50% of patients with primary colorectal carcinoma develop liver metastases. Understanding the genetic differences between primary colon cancer and their metastases to the liver is essential for devising a better therapeutic approach for this disease. We performed whole exome sequencing and copy number analysis for 15 triplets, each comprising normal colorectal tissue, primary colorectal carcinoma, and its synchronous matched liver metastasis. We analyzed the similarities and differences between primary colorectal carcinoma and matched liver metastases in regards to somatic mutations and somatic copy number alterationss. The genomic profiling demonstrated mutations in APC(73%), KRAS (33%), ARID1A and PIK3CA (6.7%) genes between primary colorectal and metastatic liver tumors. *TP53* mutation was observed in 47% of the primary samples and 67% in liver metastatic samples. The grouped pairs, in hierarchical clustering showed similar somatic copy number alteration patterns, in contrast to the ungrouped pairs. Many mutations (including those of known key cancer driver genes) were shared in the grouped pairs. The ungrouped pairs exhibited distinct mutation patterns with no shared mutations in key driver genes. Four ungrouped liver metastasis samples had mutations in DNA mismatch repair genes along with hypermutations and a substantial number of copy number alterations. Our results suggest that about half of the metastatic colorectal carcinoma had the same clonal origin with their primary colorectal carcinomas, whereas remaining cases were genetically distinct from their primary carcinomas. These findings underscore the need to evaluate metastatic lesions separately for optimized therapy, rather than to extrapolate from primary tumor data.

## Introduction

The emerging concept of polyclonality is gaining importance in cancer biology [1]. The monoclonal evolution of a tumor from a single cancer cell has been extensively studied, and is generally considered to involve the selective clonal expansion of dominant tumor clones. More recently, the alternative concept of polyclonal evolution has emerged. This model consists of two key concepts: the self-seeding hypothesis and the mutator phenotype model. The former proposes that tumor clones leave the primary site, enter systemic circulation via tumor vasculature, and colonize a distant site, thereby establishing a new subpopulation [2], [3]. The mutator phenotype model proposes a small number of highly diverse tumor cell clones (polyclonal) instead of a few competing clonal subpopulations; in fact, several solid tumor types, including colon cancers, have been suggested to be highly polyclonal [4].

Identifying the origin of cancer is pivotal in understanding the genetic events involved in tumor initiation and progression [5]. As with primary cancers, metastases can also have either a single or polyclonal origin [6], [7]. Generally, metastases carry similar mutations to those of the primary cancers from which they originate, but additional mutations occur after transformation [6], [8]. The continual, and often accelerating incidence of mutations results in genetic heterogeneity between primary and metastatic cancers; this mostly increases the resistance to therapy in the latter, which is the predominant cause of cancer-related death worldwide [6], [9].

Colorectal carcinoma (CRC) is the third most common malignancy and the second leading cause of cancer deaths in many countries [10], [11]. Nearly 50% of CRC patients develop colorectal liver metastasis (CLM) [12]. Without treatment, patients with CLMs have a median survival of only 5–10 months, with less than 0.5% surviving beyond 5 years [13]. Several studies have addressed the clonal origin and genetic heterogeneity of CRCs [14], [15]. No clear consensus has emerged from this as, although one report concluded that tumors mainly originate from a single clone [15], the results of other studies suggested that the majority of tumors have a polyclonal origin [16], [17]. Recently, whole genome sequencing of matched primary and metastatic acral melanoma has also revealed considerable genetic heterogeneity between the primary and metastatic tumors, as evidenced by *de novo*, non-synonymous single nucleotide variation [18]. Pancreatic cancer metastases have also been sequenced to evaluate the clonal relationships between primary and metastatic cancers, leading to the identification of clonal populations that gave rise to distant metastases [19], [20]. It is therefore vital to understand different concepts relating to the origin of cancer and the genetic heterogeneity between a primary tumor and its distant metastases for developing effective therapeutic strategies [21], [22].

In order to assess the polyclonality and genetic heterogeneity in CRC, we evaluated the genetic and clonal relationship between primary CRCs and their matched CLMs by performing targeted exome sequencing and high resolution copy number variation (SCNA) analysis of 15 triplets of normal colorectal tissue, primary CRC, and matched CLM samples. Our results provide valuable insights into the clonal relationship and genetic differences between primary CRCs and their matched CLMs, and will consequently help in defining potential targets for systemic therapies.

## Results

### Patient cohort description

The median age of patients in the study was 61 (Table 1). The cohort included 1 T2 stage, 10 T3 stage, and 4 T4 CRC tumors, all of which are primary resection specimens. Six patients had single hepatic metastasis while 9 patients had two or more hepatic metastases at resection. Clinical and histo-pathological information for the cohort set used in the study is provided in Table 1.

**Table 1:** Patient characteristics (N = 15).

Variables	Number (%)
**Median Age**	
**Range**	61 (43–81)
**Gender**	
**Male: Female**	11∶4
**Primary site**	
**Colon**	11 (73.0%)
**Rectum**	6 (27.0%)
**Primary T stage site**	
**T2**	1 (7%)
**T3**	10 (67%)
**T4**	4 (27%)
**Primary N stage site**	
**N0**	2 (13%)
**N1**	5 (33%)
**N2**	8 (53%)
**Tumor location in liver**	
**Unilobar**	12 (80%)
**Bilobar**	3 (20%)
**CEA before hepatectomy**	
**<5.0 ng/dL**	6 (40%)
**≥5.0 ng/dL**	9 (60%)
**CA 19-9 before hepatectomy**	
**<35 U/mL**	11 (73%)
**≥35 U/mL**	4 (27%)
**Type of hepatic resection**	
**Minor**	9 (60%)
**Major**	6 (40%)
**Cell type**	
**Well/Moderately differentiated**	13 (87%)
**Poorly differentiated**	2 (13%)
**Number of hepatic metastasis**	
**Single**	6 (40%)
**2 or more**	9 (60%)
**Completeness of liver resection**	
**R0**	15 (100%)
**R1**	0 (0%)

### SCNA analysis

We performed SCNA analysis on 15 triplets of normal colorectal tissue, CRC, and CLM samples. Using paired analysis (i.e., normal colorectal tissue vs. CRC or normal colorectal tissue vs. CLM), we identified somatic SCNAs in either the CRC or CLM samples (Table 2 and Figure S1). CRC and CLM pairs from 11 patients showed a similar number of SCNAs (Table 2); however, the remaining 4 pairs (#250, #262, #526, and #721) showed a substantial increase in the overall number of SCNAs in the CLM samples, especially those involving homozygous copy loss and loss of heterozygosity (LOH) (Table 2).

**Table 2:** Total number of SCNAs and somatic mutations in CRC-CLM pairs.

SampleID	Case	One copy gain	One copy loss	High copy gain	Homozygous loss	LOH	Total SCNA number	Somatic mutations
**185**	CRC	837	271	135	92	17	1335	101
	CLM	829	65	0	0	1	894	34
**250**	CRC	2538	1728	110	0	12	4376	102
	*CLM	3334	2611	289	132	164	6366	890
**262**	CRC	1241	875	10	0	0	2126	95
	*CLM	1121	1601	72	42	146	2836	916
**278**	CRC	745	378	27	139	180	1289	72
	CLM	1102	533	24	132	62	1791	65
**353**	CRC	289	67	9	7	0	372	66
	CLM	1634	1089	0	0	0	2723	77
**381**	CRC	2339	578	2	0	0	2919	16
	CLM	683	392	15	0	0	1090	84
**413**	CRC	389	643	4	3	0	1039	55
	CLM	351	211	23	0	0	585	63
**503**	CRC	1493	470	19	0	1	1982	60
	CLM	1103	2187	26	0	3	3316	65
**509**	CRC	1290	508	0	0	0	1798	71
	CLM	240	98	14	0	6	352	98
**523**	CRC	792	256	16	0	1	1064	90
	CLM	148	84	13	0	7	245	101
**526**	CRC	107	107	0	0	0	214	44
	*CLM	167	281	25	43	145	516	971
**627**	CRC	1863	1878	0	1	1	3742	44
	CLM	811	648	0	0	0	1459	39
**707**	CRC	658	1070	0	0	0	1728	93
	CLM	1276	565	235	4	80	2080	81
**718**	CRC	979	330	14	0	0	1323	58
	CLM	1522	327	113	45	0	2007	69
**721**	CRC	538	674	0	0	20	1212	113
	*CLM	624	307	44	62	160	1037	819

*These four CLM samples are hypermutated and also have higher number of SCNAs than other CLMs.

**Figure 1:**
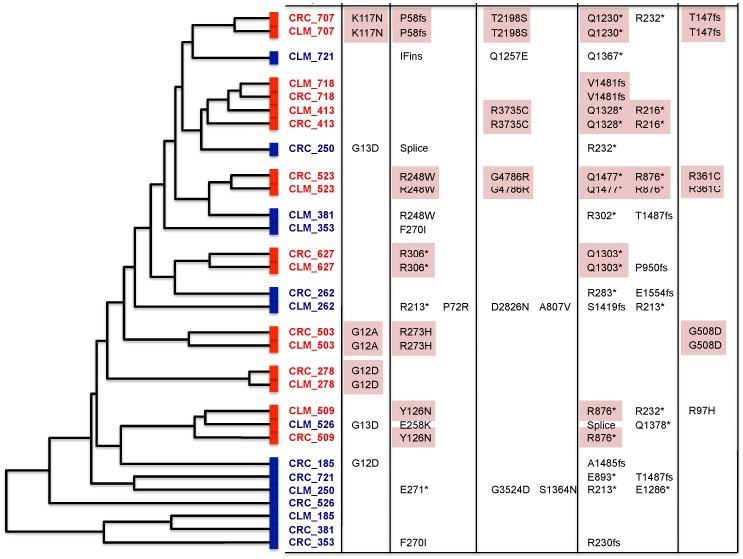
Unsupervised hierarchical clustering analysis of SCNA data. In 8 CRC-CLM pairs (53%), each pair is most closely related in the hierarchical tree (Red); in 7 CRC-CLM pairs (47%), each pair is remotely related, indicating that primary CRCs and their matched CLMs have distinct genetic features (blue). The somatic mutations in cancer related genes are also mentioned.

We performed unsupervised hierarchical clustering of the somatic SCNA data from 15 pairs of CRC and CLM samples in order to evaluate the genetic diversity between the primary and metastatic CRCs. Unsupervised hierarchical clustering of SCNA data has been used previously to determine the genetic relationships between primary and metastatic cancers [23]. The present analysis was based on the assumption that genetically similar CRCs and their matched CLMs will be closely related in hierarchical clustering. Fifty-three percent (8 of 15) of the primary CRCs were closely related to their matched CLMs, indicating clonal and genetic similarity in these CRC-CLM pairs. The remaining 47% (7 of 15) of the CRC-CLM pairs were only distantly related, suggesting distinct genetic relationships between these CRCs and their matched CLMs (Figure 1).

Of note. SCNA patterns of 8 closely related pairs were similar while those of the 7 distantly related CRC-CLM pairs were distinct (Figure S2). We also calculated and compared the average numbers of one copy gains, one copy losses, high copy gains, homozygous losses and LOH between grouped and ungrouped CRC-CLM pairs (Figure S3). Thorough comparison of hierarchical clustering and genetic similarities are described in the next section of the results.

### Exome sequencing

We performed whole exome sequencing on 15 triplets of normal colorectal tissue, CRC, and CLM samples. Using a PCR-based microsatellite assay, we confirmed that all 15 primary CRC samples were microsatellite-stable. Mutations were detected and filtered according to our in-house bioinformatics workflow (Figure S4). In total, 1079 and 4366 mutations were identified in the CRC and CLM samples, respectively (Table S1). The mutation spectra observed in the CRC and CLM samples were consistent with previous observations in solid tumors and were not significantly different between the CRC and CLM samples (Figure S5) [24].

### Hypermutation and SCNA

**Figure 2:**
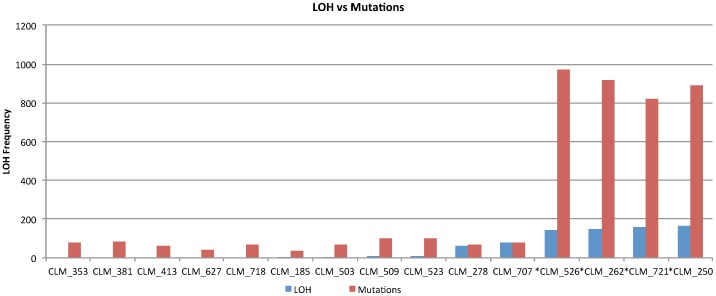
Comparison of mutation and LOH frequency in 15 CLM samples. Samples with hypermutation also contain high LOH frequency (**P-value** = 2.782e-09).

Four CLM samples (#250, #262, #525, and #721) were hypermutated (Table S2). In order to identify the cause of hypermutation, we evaluated mutations in the DNA polymerase genes (POLN, POLL, POLQ, POLH, POLE, POLD1 and POLG) and DNA mismatch repair pathway genes, including *MLH1*, *MLH3*, *MSH2*, *MSH6*, *PMS2*, *PMS6*, *ERCC2*, *ERCC5*, *ERCC6*, *MUTYH*, *RAD9A*, *EXO1*, *SLX4*, *ATR*, and *BLM*. The results revealed that only the four hypermutated CLM samples (#250, #262, #526, and #721) had one or more missense mutations in the DNA polymerase genes and DNA mismatch repair genes (Table S3). These four hypermutated samples also showed a high degree of SCNAs compared to their matched primary CRCs (Table 2), and it was particularly noteworthy that hypermutation was significantly associated with a high frequency of LOH (*P* = 2.782×10^−9^) (Figure 2).

### Somatic mutation profiles and significantly altered pathways

**Figure 3:**
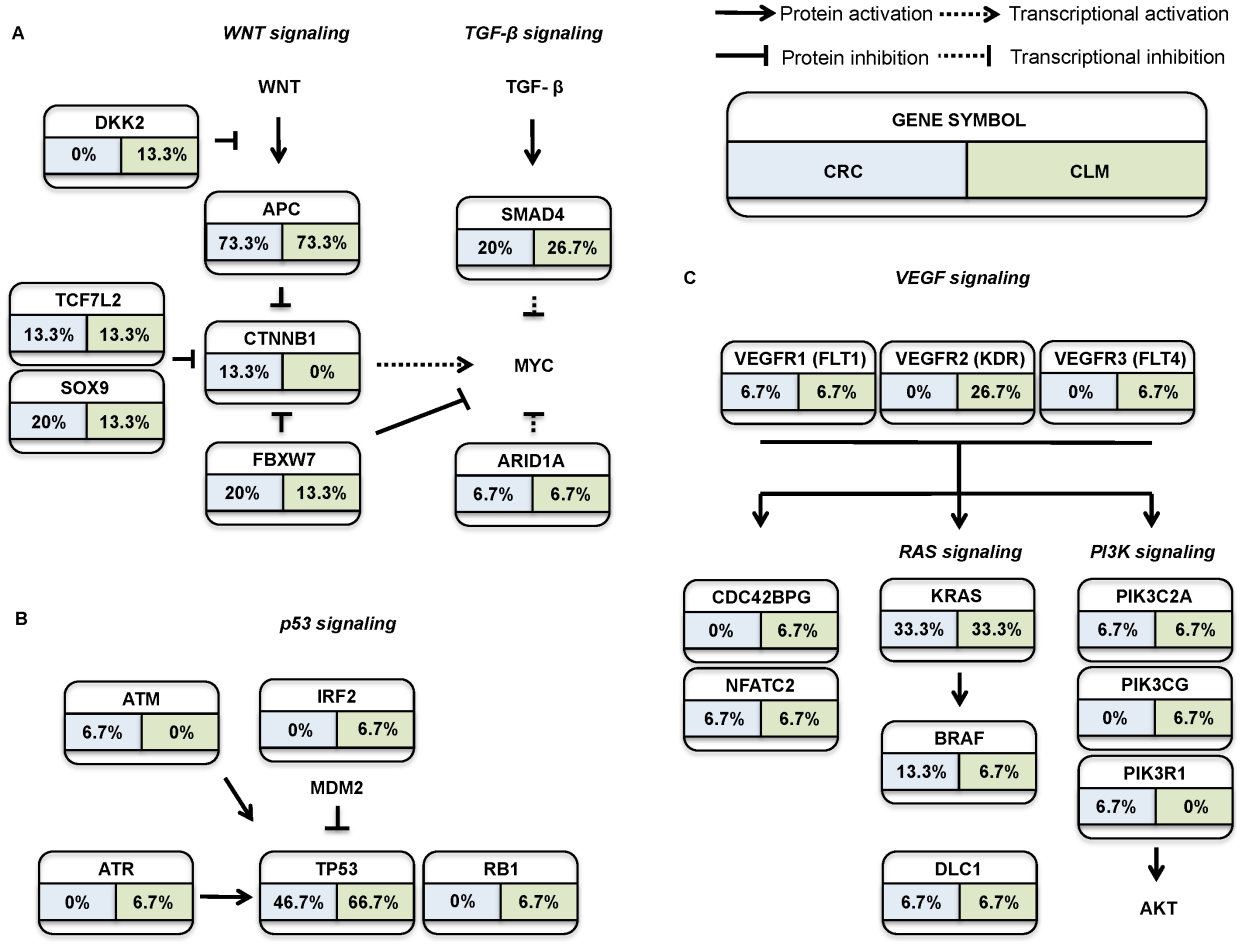
Major signaling pathways altered in CRCs and CLMs.

We found that 2,224 genes had at least 1 non-synonymous, splicing, or frameshift mutation in the CRCs or CLMs (Table S5). This data was then used to investigate the mutational status of the major signaling pathways altered in CRC (i.e., those centered on P53, Wnt, TGF-Beta, and VEGF), by comparing the frequencies with which the genes involved in these pathways were mutated (Figure 3). This revealed that *APC* was mutated in 73% of both the CRC and CLM samples, *TP53* in 47% of the CRC samples and 67% of the CLM samples, and *KRAS* in 33% of both the CRC and CLM samples. *SMAD4*, *FAT4*, and *BRAF* were also mutated in the CRC and CLM samples with varying frequencies. In the VEGF signaling pathway, we found mutations in the *KDR* (0% and 27%), *FLT1* (7% and 7%), and *FLT4* (0% and 7%) genes of the CRCs and CLMs, respectively (Figure 3). Mutations in VEGF pathway genes were mainly confined to the CLM samples, the most striking example of which being the *KDR* gene which was only mutated in the hypermutated CLM samples (Table 3).

**Table 3:** Mutations in VEGFR pathway genes.

Mutation	Gene Name	CRC	CLM
**chr4:55972974**	KDR	.	250, 526, 721
**chr4:55979558**	KDR	.	262
**chr13:28964203**	FLT1	721	.
**chr13:29001960**	FLT1	.	721
**chr5:180038416**	FLT4	.	262

All four hypermutated CLM samples had mutations in KDR gene.

### Evaluating genetic relationships on the basis of mutations and SCNA

To evaluate genetic relationships between the 15 pairs of CRC and CLM samples, we evaluated whether the same genotypic changes and mutations in major cancer driver genes occurred in each pair. This evaluation was based on the assumption that CRC and CLM pairs with similar genetic alterations are likely to share mutations in the major driver genes. In eight cases, the CRC-CLM pair did indeed share mutations in key CRC-related genes (*APC*, *KRAS*, *TP53*, *SMAD4*, *BRAF*, and *FAT4*) (Table S4). In these pairs, no significant difference was observed in the number of mutations between the CRC and CLM samples (*P* = 0.28) (Table 4). Conversely, in the remaining seven cases, none of the CRC-CLM pairs shared a mutation in key CRC-related genes; however, the total number of mutations differed significantly between the CRC and CLM samples (*P*<0.05) (Table 4 and Table S4).

**Table 4:** Percentage of shared point mutations in CRC-CLM pairs.

Sample ID	Hierarchical clustering*	CRC Mutations		CLM Mutations		Shared point mutations (%)		
**185**	Remotely related		101		34		0	
**250**	Remotely related		102		890		0	
**262**	Remotely related		95		916		0.2	
**353**	Remotely related		66		77		52.1	
**381**	Remotely related		16		84		3.1	
**526**	Remotely related		44		971		0	
**721**	Remotely related		113		819		0.1	
**278**	Most closely related		72		65		53.9	
**413**	Most closely related		55		63		43.9	
**503**	Most closely related		60		65		38.9	
**509**	Most closely related		71		98		30	
**523**	Most closely related		90		101		36.4	
**627**	Most closely related		44		39		45.6	
**707**	Most closely related		93		81		35.9	
**718**	Most closely related		58		69		46	

*It indicates whether primary CRCs and their matched CLMs are most closely related in the hierarchical tree (i.e., whether they are genetically most similar based on SCNA data).

Importantly, concordance analysis of the mutation data reconciled with the SCNA data analysis. All CRC-CLM pairs, which were closely related in the unsupervised hierarchical clustering of SCNA data, shared mutations in key CRC-related genes (53%). However, the seven CRC-CLM pairs that were only remotely related in the hierarchical clustering of the SCNA data, did not share a mutation in key CRC-related genes (47%).

Taken together, these findings indicate that in 53% of the cases, each CRC-CLM pair had similar genetic alterations, whereas those in the remaining 47% of the cases had distinct genetic alterations.

## Discussion

By comparing the SCNA data and mutation profiles of 15 paired CRC and CLM samples, we found that approximately half of them, showed genetic heterogeneity with respect to their corresponding primary CRC. To the best of our knowledge, this is the first comprehensive study to use genomic profiling of primary CRCs and their matched metastases and to define the distinct features of the metastatic lesions in terms of their mutation and SCNA profiles.

Fifty-three percent of the CRC-CLM pairs in the clustered group shared a high number of mutations, including some in the *APC*, *KRAS*, *TP53*, and *SMAD4* genes (Table 4, Figure 1). The presence of many shared mutations (30–65%) indicated that somatic mutations may accumulate within the microenvironment of a primary cancer before disseminating to their metastatic sites, something commonly referred to as the linear progression model of tumor evolution [22]. The remaining 47% of the CRC-CLM pairs, which were grouped independently of each other, showed significant differences in their mutation profiles and SCNA data. They had no shared mutations in cancer initiating genes and no significant differences in SCNA profiles (Figure 1). The distinct relationship and prominent genetic heterogeneity in these pairs indicate that the CLMs might have originated from a group of genetically distinct primary CRC clones interacting in close proximity with polyclonal model of tumor progression.

Six CRC-CLM pairs had somatic KRAS mutations in at least one sample. Three pairs had the same KRAS mutations, and the other three did not. Therefore, the discordance rate of KRAS mutation between CRC-CLM pairs was 50% (3/6) (Figure 1 and Table S4). Knijn and colleagues [25] reported high concordance rate of KRAS mutations between primary CRC and CLM tumors. In contrast, a series of studies have demonstrated high discordance rate, between CRC-CLM pairs, of KRAS mutations ranging from 8%–60% [26]–[28]. In our study, those three CRC-CLM pairs with discordant KRAS mutation status were also clustered distinctly by SCNA analysis. The discordance of KRAS mutation, along with distinct SCNA clustering patterns, between these CRC-CLM pairs supports our hypothesis that primary CRCs and their corresponding CLMs may have different clonal origins in these samples.

The polyclonal tumor progression model can help direct therapeutic strategies [4]. The major disadvantage of a monoclonal tumor origin model is the assumption that most of the initial events that led to the primary cancer will also be found in the metastasized cells, which overlooks the possibility that small populations of tumor cells with distinct genetic characteristics in close proximity to each other may be responsible for the metastasis[4]. Such metastatic cells, which originate from primary tumors, might have a different response to therapy.

Hypermutation caused by the loss of DNA mismatch repair activity is termed MSI [29]. We found that four CLM samples were instable microsatellites, resulting in hypermutations. The *KDR* gene, a significant prognostic marker in colorectal carcinoma [30], was mutated only in the hypermutated samples. It is also noteworthy that there was an apparent relationship between hypermutation and chromosomal instability. Recently, a distinct copy number status of the DNA mismatch repair gene *MLH1* was shown to be associated with elevated levels of mutation in pancreatic cancer [31]. Previous studies showed contradicting evidences about MSI tumors and chromosome instability in colorectal cancers. Some studies reported that MSS tumors show higher rate of chromosome instability than MSI tumors [32]–[35]. Other studies reported substantial overlap between MSI tumors and chromosome instability [32], [35]–[38]. Of note, all these studies were done using primary CRC samples, and the relationship of MSI and chromosome instability in CLM samples is yet to be revealed. We found that MSI tumors were associated with a large number of gene deletions/amplifications and increased frequency of LOH, in other words, chromosomal instability in CLM tumors. Further research is required to reveal the relationship between MSI, chromosome instability and metastasis of primary CRCs.

Angiogenesis is regulated principally by interactions between vascular endothelial growth factors and VEGF receptors and play a central role in cancer growth and metastasis [39], [40]. Several studies have reported the genetic polymorphism of the KDR gene implicating the risk of coronary artery diseases [41], [42]. However, the clear role of individual KDR SNPs and their physiological functions in cancer progression and prognosis remains unknown. In the current study, all of the patients with KDR SNPs (i.e., rs187037 and rs2305948) had recurrence after curative resection of CRC and liver (p = 0.925; data not shown). However, due to small sample KDR mutation was not statistically significant. A larger number of samples are needed to validate the KDR mutation and its characteristic role in tumor recurrence.

The mean survival time of patients with metastatic CRC has increased from 6–8 months to more than 2 years due to the emergence of targeted treatment and improved surgical resections. Nevertheless, the therapeutic option for non-responders to oxaliplatin- or irinotecan-based chemotherapy, with or without cetuximab or bevacizumab, is very limited. Hence, better treatment strategies for metastatic CRC have to be developed. An emerging body of evidence suggests that primary CRC may present as polyclonal in nature and that the resulting metastases might therefore have a genetically different from the majority of the primary tumor. In such cases, the biology and genetic profile of the primary tumor may be significantly different from the metastases. This would be an important concern in targeted, personalized therapy. Our results suggest that the mutational profiles of approximately 50% of metastatic liver tumors might be different from that of the primary tumor, which underscores the need to evaluate metastatic sites separately for identification of potential targets for systemic therapy.

## Materials and Methods

### Study population

Between June 2009 and June 2011, 53 patients underwent curative resection of CRC and liver metastasis at Gachon University Gil Hospital (Incheon, South Korea). The criteria for inclusion in this study were as follows: (1) hepatic metastasis from CRC confirmed by spiral abdominopelvic computed tomography; (2) liver metastasis as the first manifestation of M1 disease without any documented disseminated disease, as determined by preoperative imaging; (3) no prior history of neoadjuvant chemoradiation or chemotherapy, including molecular targeted agents; (4) curative resection performed for both primary colorectal and liver lesions; (5) the resected specimens should be synchronous tumors (simultaneous resection, *n* = 11; two-stage resection within 6 months, *n* = 4); and (6) microsatellite stable primary CRCs. We selected 15 patients with CRC and matched liver metastasis based on these inclusion criteria. The basic characteristics of the patients are shown in Table 1. All tumors were reviewed by a single pathologist, and only specimens with >70% tumor content were included in the analysis. The study protocols were approved by the Institutional Review Board of Gachon University Gil Hospital (IRB approval number: GIRBA 2535). Written informed consent was required from all participants. Information, such as sex, age, tumor stage, was extracted from the clinical database for this cohort.

### PCR-based microsatellite assay

A set of microsatellite markers consisting of two mononucleotide repeat markers (BAT25 and BAT26) and three dinucleotide repeat markers (D2S123, D5S346, and D17S250), as recommended by the National Cancer Institute Consensus Group, were used to determine tumor the microsatellite instability (MSI) status. Aliquots containing 50 ng DNA were amplified in 20-µL reaction mixtures containing 2 µL of 10× buffer (Roche, Mannheim, Germany), 1.7–2.5 mmol/L MgCl_2_, 0.3 µM each primer pair, 250 µM deoxynucleotide triphosphates, and 2.5 U DNA polymerase (Roche, Mannheim, Germany). PCR was performed with an initial denaturation step of 94°C for 5 min, followed by 30 cycles of 1 min at 94°C, 1 min at 55°C, and 1 min at 72°C and a final extension step of 10 min at 72°C. The samples were analyzed on an ABI Prism 3100 Genetic Analyzer using 0.7 µL of amplified sample combined with 0.3 µL of GeneScan 500 Size Standard and 9 µL of HiDi Formamide according to the manufacturer's guidelines (Applied Biosystems, Foster City, CA, USA). Data were analyzed using ABI Prism 3100 Data Collection software (Applied Biosystems, Foster City, CA, USA).

### DNA extraction, library preparation, and targeted exome sequencing

DNA was extracted using a DNeasy Blood & Tissue Kit (QIAGEN, Valencia, CA, USA). DNA quality was checked by 1% agarose gel electrophoresis, and DNA concentration was measured using a PicoGreen dsDNA Assay (Invitrogen, Carlsbad, CA, USA). SureSelect sequencing libraries were prepared according to the manufacturer's instructions (Agilent SureSelect All Exon Kit 38 Mb; Agilent Technologies, Santa Clara, CA, USA) using a Bravo automated liquid handler. The quality of the amplified libraries was verified by capillary electrophoresis (Bioanalyzer; Agilent Technologies, Santa Clara, CA, USA), after which paired-end DNA sequences were obtained from the libraries using the Illumina HiSeq platform (Illumina, San Diego, CA, USA).

### Bioinformatics analysis

Sequence data were aligned to the human reference genome GRCh37 (http://www.ncbi.nlm.nih.gov/projects/genome/assembly/grc/human/index.shtml) using the Burrows-Wheeler Aligner [43] with default parameters. We sequenced at the average depth of 52.44X for targeted regions. The PCR duplicates were removed using the Picard algorithm (http://picard.sourceforge.net). We performed realignment and quality recalibration for the sequenced data using the Genome Analysis Toolkit (GATK) [44]. After alignment, we used Varscan [45], Strelka [46], and Mutect [47] to call mutations, including insertions and deletions (indels), for each chromosomal position and also used GATK [44] for indel detection with 15 triplet specimens consisting of normal colorectal tissue, primary CRC, and matched CLMs. We annotated the mutations using ANNOVAR [48] with the Ensembl Gene annotation database for human genome build 37 (http://www.ensembl.org/) and searched for matches in the dbSNP137 (http://www.ncbi.nlm.nih.gov/projects/genome/assembly/grc/human/index.shtml), 1000 genomes data [49], and COSMIC database [50]. We filtered the mutations from the targeted regions and selected non-synonymous, synonymous, gain or loss of the stop codon, frameshift indels, non-frameshift indels, and splicing site mutations.

### SCNA analysis

The single nucleotide polymorphism (SNP) array of CytoScan™ HD (Affymetrix, Inc., Santa Clara, CA, USA) was used. SCNA analysis of the CytoScan™ HD Array was performed using BioDiscovery Nexus Copy Number 6.1 (http://www.biodiscovery.com/software/nexus-copy-number/) software. The SNP-Fast Adaptive States Segmentation Technique 2 segmentation algorithm was used with default parameters.

### Clustering

Complete linkage hierarchical clustering was performed to evaluate the concordance between the primary CRC and CLM samples. Average and single linkage hierarchical clustering were also applied; however, all clustering methods yielded similar results. Paired *t*-test and two-sample *t*-test were used for statistical analyses, and *P*<0.05 was considered to indicate statistical significance.

### Data Link

Whole exome sequencing data: Sequence Retrieve Archive (SRA) accession number is SRP034161.

Cytoscan array data: GEO accession number is GSE53799.

## Supporting Information

Figure S1 Workflow for copy number variation (CNV) analysis.
(TIF)

Figure S2 SCNA patterns of CRC-CLM pairs. Gains are represented in blue, losses in red and LOH in brown. 8/15 CRC-CLM grouped pairs showed high similarity in SCNA patterns compared to the rest of the 7/15 CRC-CLM ungrouped pairs.
(TIF)

Figure S3 Average SCNA frequencies of grouped and ungrouped CRC-CLM pairs. (A) High copy gains, homozygous losses and LOH are highly variable in ungrouped CRC-CLM pairs. High copy gains in grouped CRC-CLM also showed variability. (B) One copy losses in ungrouped CRC-CLM pairs showed variability.
(TIF)

Figure S4 Workflow for whole exome sequencing analysis.
(TIF)

Figure S5 Mutation spectra of CRCs and CLMs. (A) Mutation spectrum of CRCs and their matched CLMs except hypermutated samples. (B) Mutation spectrum of four hypermutated CLM samples.
(TIF)

Table S1 Number of somatic mutations in CRCs and CLMs.
(DOCX)

Table S2 Total number of mutations in each CRC-CLM pairs.
(DOCX)

Table S3 Mutational status of DNA mismatch repair pathway genes and DNA polymerase genes in CRCs and CLMs.
(DOCX)

Table S4 Mutational concordance between CRC and CLM pairs. All closely related CRC-CLM pairs in hierarchical clustering shared mutations in key CRC-related genes.
(DOCX)

Table S5 Mutations and mutated genes in the CRCs and CLMs (the table is provided as excel files).
(XLSX)
