# Correction: Comparative Genomic Analysis of Primary and Synchronous Metastatic Colorectal Cancers

**DOI:** 10.1371/journal.pone.0117753

**Published:** 2015-01-21

**Authors:** The PLOS ONE Staff

There are a number of errors in S5 Table of the published article. The correct S5 Table can be viewed here.

## Supporting Information

S5 Table Mutations and mutated genes in the CRCs and CLMs. (XLSX)

## References

[1] Lee SY, Haq F, Kim D, Jun C, Jo H-J, Anh S-M, et al (2014) Comparative Genomic Analysis of Primary and Synchronous Metastatic Colorectal Cancers. PLoS ONE 9(3): e90459 doi:10.1371/journal.pone.0090459 24599305 10.1371/journal.pone.0090459 PMC3944022

