# Supplementary figures and images for: Comparative Genomic Analysis of Primary and Synchronous Metastatic Colorectal Cancers
Source: PLoS One. 2014 Mar 5;9(3):e90459. doi: 10.1371/journal.pone.0090459 (PMC3944022; doi:10.1371/journal.pone.0090459)

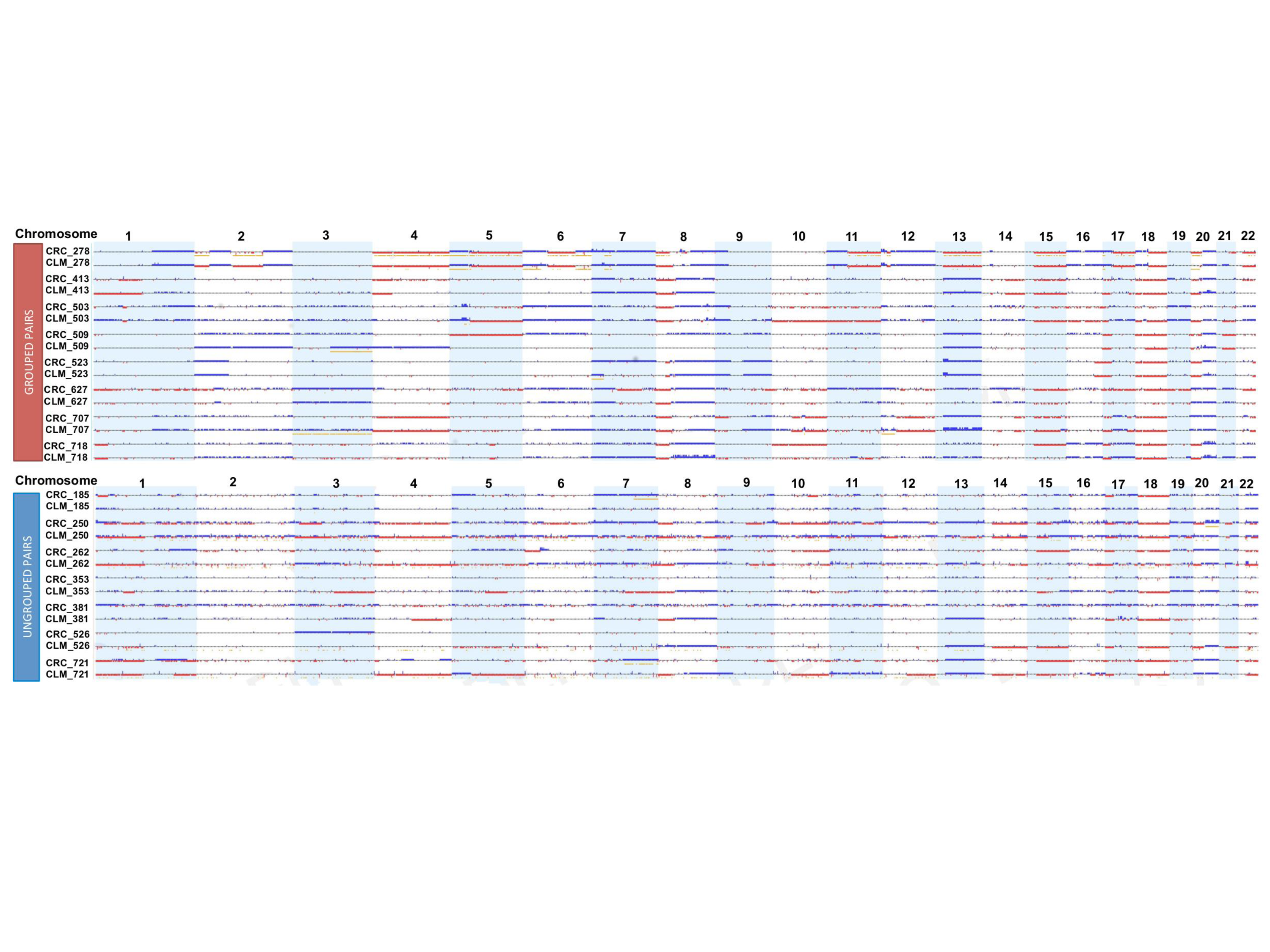

Supplement: Figure S2 — SCNA patterns of CRC-CLM pairs. Gains are represented in blue, losses in red and LOH in brown. 8/15 CRC-CLM grouped pairs showed high similarity in SCNA patterns compared to the rest of the 7/15 CRC-CLM ungrouped pairs. (TIF) [file pone.0090459.s002.tif]

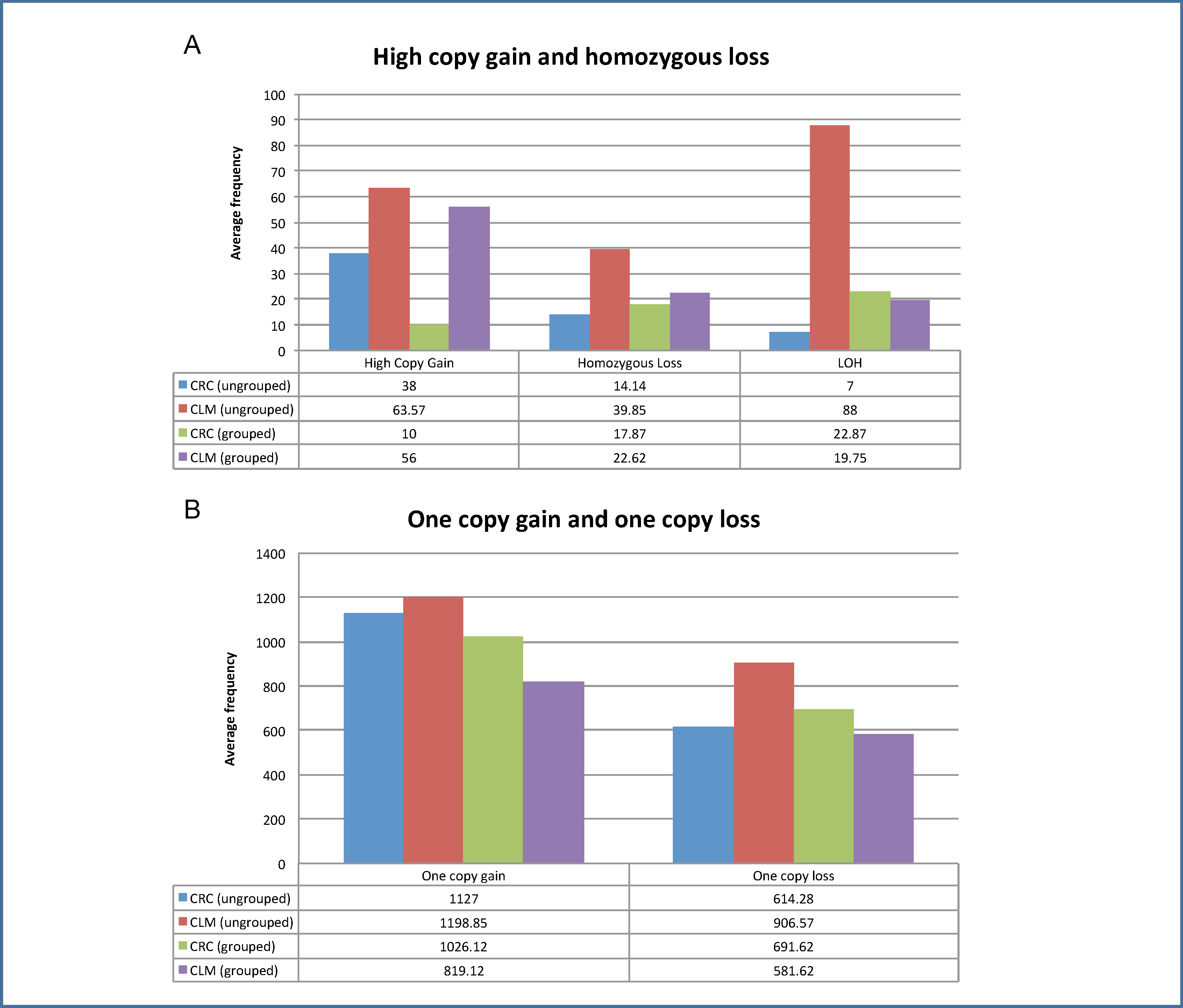

Supplement: Figure S3 — Average SCNA frequencies of grouped and ungrouped CRC-CLM pairs. (A) High copy gains, homozygous losses and LOH are highly variable in ungrouped CRC-CLM pairs. High copy gains in grouped CRC-CLM also showed variability. (B) One copy losses in ungrouped CRC-CLM pairs showed variability. (TIF) [file pone.0090459.s003.tif]

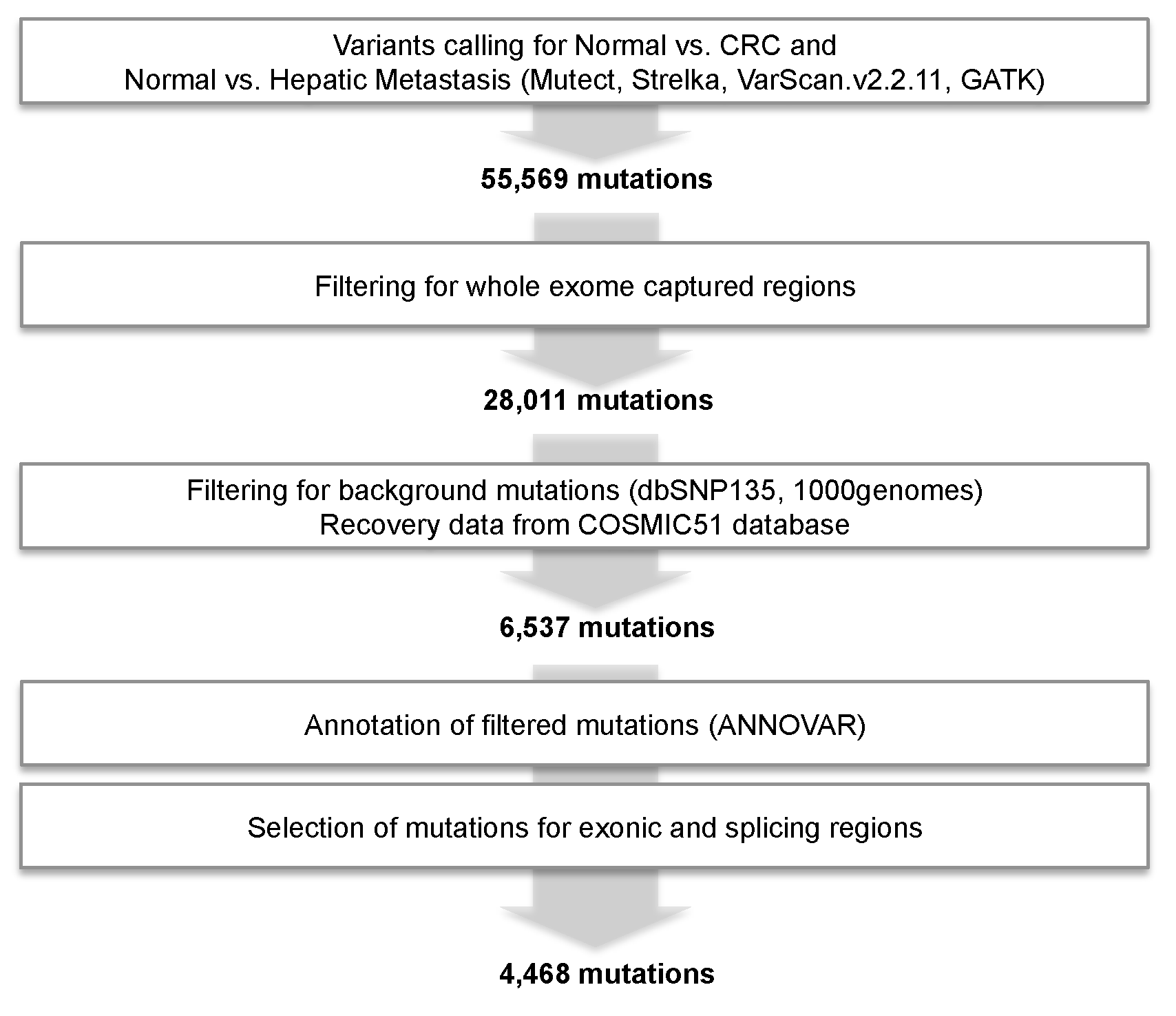

Supplement: Figure S4 — Workflow for whole exome sequencing analysis. (TIF) [file pone.0090459.s004.tif]

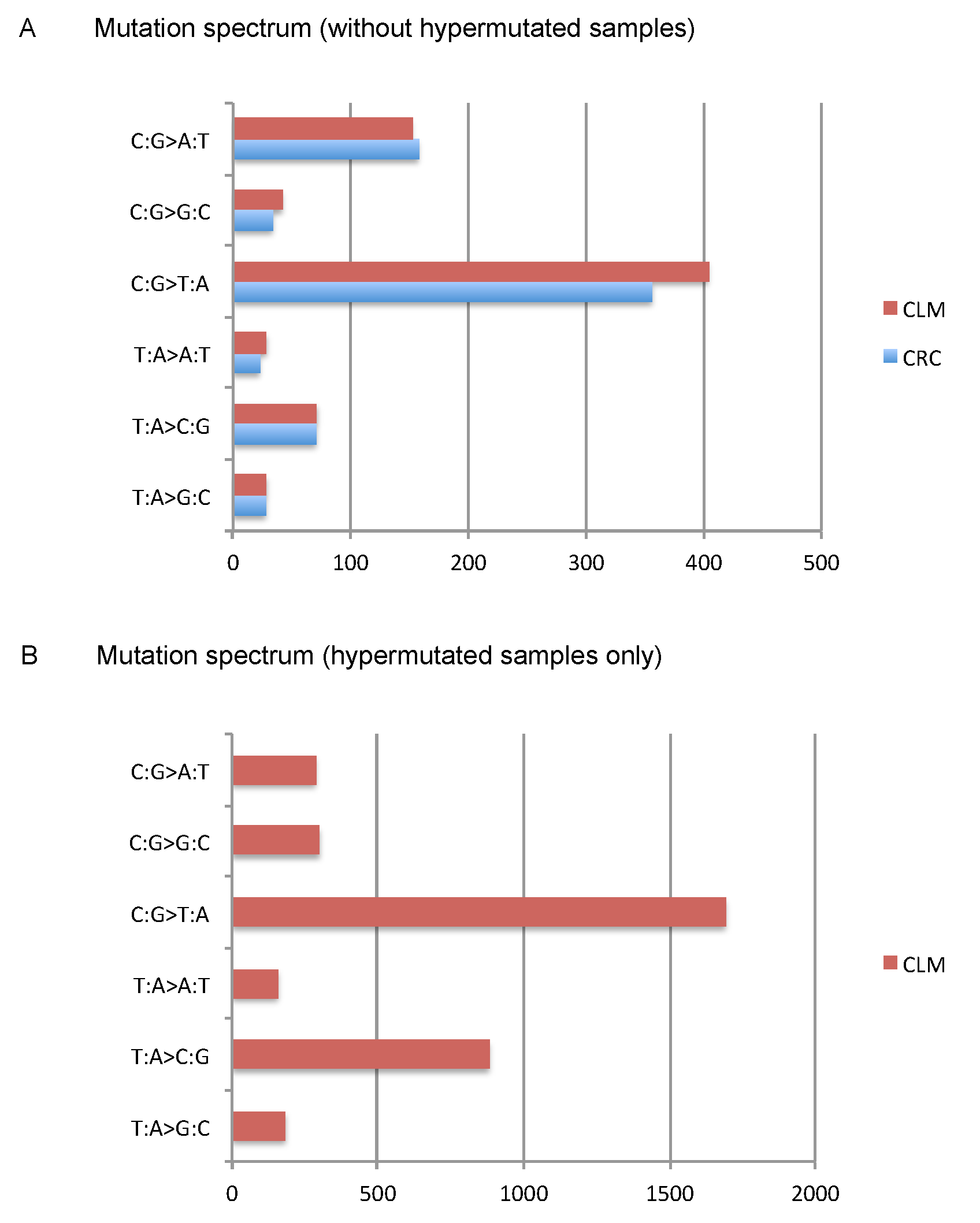

Supplement: Figure S5 — Mutation spectra of CRCs and CLMs. (A) Mutation spectrum of CRCs and their matched CLMs except hypermutated samples. (B) Mutation spectrum of four hypermutated CLM samples. (TIF) [file pone.0090459.s005.tif]
