# Supplementary material for: Comparative Genomic Analysis of Primary and Synchronous Metastatic Colorectal Cancers
Source: PLoS One. 2014 Mar 5;9(3):e90459. doi: 10.1371/journal.pone.0090459 (PMC3944022; doi:10.1371/journal.pone.0090459)
Supplement: Table S1 — Number of somatic mutations in CRCs and CLMs. (DOCX) [file pone.0090459.s006.docx]

**Table S1.** Number of somatic mutations in CRCs and CLMs.

| Types of mutations | Number of mutations | | | | Total (%) |
| --- | --- | --- | --- | --- | --- |
|  | CRC | (%) | CLM | (%) |  |
| Non-synonymous | 656 | 60.8 | 2,397 | 54.9 | 56.1 |
| Synonymous | 289 | 26.8 | 1,704 | 39 | 36.7 |
| Stop gain | 49 | 4.5 | 88 | 2 | 2.5 |
| Stop loss | 0 | 0 | 2 | 0 | 0 |
| Splicing site | 19 | 1.8 | 40 | 1 | 1 |
| Frame shift deletions | 28 | 2.6 | 55 | 1.3 | 1.5 |
| Frame shift insertions | 29 | 2.7 | 37 | 0.8 | 1.2 |
| Non-frame shift deletions | 8 | 0.7 | 24 | 0.5 | 0.6 |
| Non-frame shift insertions | 1 | 0.1 | 19 | 0.4 | 0.4 |
| Total | 1,079 | 100 | 4,366 | 100 | 100 |
