# Supplementary material for: Comparative Genomic Analysis of Primary and Synchronous Metastatic Colorectal Cancers
Source: PLoS One. 2014 Mar 5;9(3):e90459. doi: 10.1371/journal.pone.0090459 (PMC3944022; doi:10.1371/journal.pone.0090459)
Supplement: Table S2 — Total number of mutations in each CRC-CLM pairs. (DOCX) [file pone.0090459.s007.docx]

**Table S2.** Total number of mutations in each CRC-CLM pairs.

| Sample ID | Case | Cluster | Ns-mutation | s- mutation | Stop gain | Stop loss | Splicing site | FS del | FS ins | nFS del | nFS ins |
| --- | --- | --- | --- | --- | --- | --- | --- | --- | --- | --- | --- |
| 185 | CRC | N | 66 | 21 | 5 | 0 | 3 | 2 | 4 | 0 | 0 |
|  | CLM |  | 23 | 9 | 0 | 0 | 1 | 2 | 0 | 0 | 0 |
| 250* | CRC | N | 62 | 27 | 7 | 0 | 2 | 1 | 3 | 0 | 0 |
|  | CLM |  | 482 | 369 | 12 | 0 | 5 | 4 | 8 | 3 | 7 |
| 262* | CRC | N | 61 | 25 | 4 | 0 | 2 | 1 | 2 | 0 | 0 |
|  | CLM |  | 490 | 374 | 16 | 0 | 7 | 7 | 8 | 8 | 6 |
| 278 | CRC | Y | 48 | 16 | 1 | 0 | 4 | 3 | 0 | 0 | 0 |
|  | CLM |  | 42 | 15 | 2 | 1 | 3 | 2 | 0 | 0 | 0 |
| 353 | CRC | N | 32 | 24 | 1 | 0 | 1 | 4 | 3 | 1 | 0 |
|  | CLM |  | 42 | 27 | 2 | 0 | 1 | 1 | 3 | 1 | 0 |
| 381 | CRC | N | 11 | 3 | 0 | 0 | 0 | 0 | 2 | 0 | 0 |
|  | CLM |  | 52 | 19 | 7 | 0 | 1 | 4 | 0 | 1 | 0 |
| 413 | CRC | Y | 34 | 12 | 3 | 0 | 2 | 0 | 2 | 2 | 0 |
|  | CLM |  | 43 | 14 | 2 | 0 | 2 | 0 | 1 | 1 | 0 |
| 503 | CRC | Y | 39 | 12 | 2 | 0 | 1 | 2 | 4 | 0 | 0 |
|  | CLM |  | 45 | 13 | 5 | 0 | 0 | 1 | 1 | 0 | 0 |
| 509 | CRC | Y | 44 | 16 | 5 | 0 | 1 | 3 | 2 | 0 | 0 |
|  | CLM |  | 62 | 22 | 8 | 0 | 1 | 3 | 2 | 0 | 0 |
| 523 | CRC | Y | 53 | 30 | 8 | 0 | 1 | 7 | 0 | 1 | 0 |
|  | CLM |  | 61 | 25 | 7 | 0 | 0 | 7 | 0 | 1 | 0 |
| 526* | CRC | N | 32 | 8 | 0 | 0 | 1 | 1 | 1 | 1 | 0 |
|  | CLM |  | 520 | 412 | 11 | 1 | 10 | 5 | 6 | 3 | 3 |
| 627 | CRC | Y | 26 | 14 | 3 | 0 | 0 | 0 | 1 | 0 | 0 |
|  | CLM |  | 20 | 12 | 3 | 0 | 1 | 0 | 2 | 1 | 0 |
| 707 | CRC | Y | 48 | 36 | 5 | 0 | 1 | 2 | 1 | 0 | 0 |
|  | CLM |  | 52 | 21 | 1 | 0 | 4 | 3 | 0 | 0 | 0 |
| 718 | CRC | Y | 33 | 18 | 2 | 0 | 0 | 1 | 1 | 2 | 1 |
|  | CLM |  | 43 | 18 | 2 | 0 | 2 | 2 | 0 | 2 | 0 |
| 721* | CRC | N | 67 | 37 | 3 | 0 | 1 | 1 | 3 | 1 | 0 |
|  | CLM |  | 421 | 358 | 10 | 0 | 4 | 14 | 6 | 3 | 3 |

Cluster indicates hierarchical clustering results (Y: clustered, N: not clustered); Ns, nonsynonymous; s, synonymous; FS del, frame shift deletion; FS ins, frame shift insertion; nFS del, non-frame shift deletion; nFS ins, non-frame shift insertion.

* Hyper-mutated sample ID in their colorectal liver metastasis.
