# Supplementary material for: Comparative Genomic Analysis of Primary and Synchronous Metastatic Colorectal Cancers
Source: PLoS One. 2014 Mar 5;9(3):e90459. doi: 10.1371/journal.pone.0090459 (PMC3944022; doi:10.1371/journal.pone.0090459)
Supplement: Table S3 — Mutational status of DNA mismatch repair pathway genes and DNA polymerase genes in CRCs and CLMs. (DOCX) [file pone.0090459.s008.docx]

**Table S3.** Mutational status of DNA mismatch repair pathway genes and DNA polymerase genes in CRCs and CLMs.

| Mutation | COSMIC entry | Gene Name | CRC | CLM | RG variant | RG type |
| --- | --- | --- | --- | --- | --- | --- |
| 3,37067240 | Y | MLH1 | . | 250 | V>D | mis |
| 12,1040408 | N | RAD52 | . | 526 | R>H | mis |
| 15,91295110 | Y | BLM | . | 262,526 | T>M | mis |
| 1,242035382 | Y | EXO1 | . | 262 | T>M | mis |
| 1,242042301 | Y | EXO1 | . | 250 | E>K | mis |
| 16,3658568 | N | SLX4 | . | 721 | P>L | mis |
| 3,142281612 | Y | ATR | . | 250 | M>T | mis |
| 4,2176454 | N | POLN | . | 262 | R>C | mis |
| 4,2195024 | N | POLN | . | 262 | M>L | mis |
| 10,103340056 | N | POLL | . | 262 | R>W | mis |
| 3,121209245 | N | POLQ | . | 526 | A>T | mis |
| 3,121251868 | N | POLQ | . | 262 | V>G | mis |
| 6,43555068 | N | POLH | . | 250 | R>H | mis |
| 12,133244226 | N | POLE | . | 250 | R>W | mis |
| 12,133253995 | N | POLE | . | 262 | A>V | mis |
| 19,50905074 | N | POLD1 | . | 250,262 | R>H | mis |
| 15,89869870 | N | POLG | . | 526 | R>L | mis |
