# Supplementary material for: Comparative Genomic Analysis of Primary and Synchronous Metastatic Colorectal Cancers
Source: PLoS One. 2014 Mar 5;9(3):e90459. doi: 10.1371/journal.pone.0090459 (PMC3944022; doi:10.1371/journal.pone.0090459)
Supplement: Table S4 — Mutational concordance between CRC and CLM pairs. All closely related CRC-CLM pairs in hierarchical clustering shared mutations in key CRC-related genes. (DOCX) [file pone.0090459.s009.docx]

**Table S4.** Mutational concordance between CRC and CLM pairs. All closely related CRC-CLM pairs in hierarchical clustering shared mutations in key CRC-related genes.

| CHR | Position | GENE | CRC | CLM |
| --- | --- | --- | --- | --- |
| chr5 | 112116592 | APC | . | 250,262 |
| chr5 | 112128143 | APC | 413 | 413 |
| chr5 | 112128185 | APC | 353 | . |
| chr5 | 112128191 | APC | 250,707 | 509 |
| chr5 | 112151204 | APC | 262 | . |
| chr5 | 112151261 | APC | . | 381 |
| chr5 | 112155042 | APC | . | 526 |
| chr5 | 112173917 | APC | 509,523 | 509,523 |
| chr5 | 112173968 | APC | 721 | . |
| chr5 | 112174140 | APC | . | 627 |
| chr5 | 112174979 | APC | 707 | 707 |
| chr5 | 112175147 | APC | . | 250 |
| chr5 | 112175198 | APC | 627 | 627 |
| chr5 | 112175273 | APC | 413 | 413 |
| chr5 | 112175390 | APC | . | 721 |
| chr5 | 112175423 | APC | . | 526 |
| chr5 | 112175548 | APC | . | 262 |
| chr5 | 112175720 | APC | 523 | 523 |
| chr5 | 112175733 | APC | 718 | 718 |
| chr5 | 112175746 | APC | 185 | . |
| chr5 | 112175751 | APC | 721 | . |
| chr5 | 112175752 | APC | . | 381 |
| chr5 | 112175951 | APC | 262 | . |
| chr12 | 25378647 | KRAS | 707 | 707 |
| chr12 | 25398281 | KRAS | 250 | 526 |
| chr12 | 25398284 | KRAS | 185,278,503 | 278,503 |
| chr17 | 7577022 | TP53 | 627 | 627 |
| chr17 | 7577120 | TP53 | 503 | 503 |
| chr17 | 7577127 | TP53 | . | 250 |
| chr17 | 7577130 | TP53 | 353 | 353 |
| chr17 | 7577509 | TP53 | . | 526 |
| chr17 | 7577539 | TP53 | 523 | 381,523 |
| chr17 | 7578212 | TP53 | . | 262 |
| chr17 | 7578554 | TP53 | 509 | 509 |
| chr17 | 7578555 | TP53 | 250 | . |
| chr17 | 7579513 | TP53 | 707 | 707 |
| chr18 | 48575096 | SMAD4 | . | 509 |
| chr18 | 48575679 | SMAD4 | 707 | 707 |
| chr18 | 48591918 | SMAD4 | 523 | 523 |
| chr18 | 48604701 | SMAD4 | 503 | 503 |
| chr4 | 126241657 | FAT4 | . | 250 |
| chr4 | 126336711 | FAT4 | 707 | 707 |
| chr4 | 126373374 | FAT4 | 413 | 413 |
| chr7 | 140439699 | BRAF | 250 | . |
| chr7 | 140481406 | BRAF | 707 | 707 |
